# Eczema as a protective factor for brain cancer: a meta-analysis

**DOI:** 10.1186/s12885-022-10471-0

**Published:** 2022-12-29

**Authors:** Yun Zhu, Yirong Teng, Shuangyan Xu, Yinde Xu, Boheng Zhu, Weimin Yan, Jie Liu

**Affiliations:** 1 Department of Dermatology and Venereology, The Sixth Affiliated Hospital of Kunming Medical University, Yuxi, Yunnan China; 2 Department of Dermatology and Venereology, The First Affiliated Hospital of Kunming Medical University, Kunming, Yunnan China; 3 Department of General Medicine, The Sixth Affiliated Hospital of Kunming Medical University, Yuxi, Yunnan China; 4 Department of Dermatology and Venereology, The Second Affiliated Hospital of Kunming Medical University, Kunming, Yunnan China

**Keywords:** Eczema, Brain cancer, Glioma, Meningioma, Acoustic neuroma, Risk

## Abstract

**Background:**

Brain cancer is one of the most aggressive cancer types owing to poor treatment effects. Epidemiological studies have demonstrated that allergies may increase the disease risk. Therefore, this study evaluated the association between eczema and the risk of various brain cancers.

**Methods:**

We systematically searched the PubMed and Embase databases from their inception until June 23, 2022. Two reviewers independently reviewed and screened the articles, extracted data, assessed the study quality, and pooled the results. Stata software was used to generate pooled odds ratios and 95% confidence intervals (CIs).

**Results:**

We included 20 studies comprising 5,117,222 patients that investigated the relationship between eczema and brain cancer. Eczema was significantly inversely associated with the risk of brain cancer (odds ratio [OR], 0.82; 95% CI, 0.77–0.87), glioma (OR, 0.53; 95% CI, 0.14–2.02), meningioma (OR, 0.74; 95% CI, 0.66–0.84), and acoustic neuroma (OR, 0.60; 95% CI, 0.41–0.88). Interesting, The strong correlation between eczema and the reduced risk of brain cancer was observed in people over 16 years old (OR, 0.79; 95% CI, 0.71–0.88), but not in those under 16 years old (OR, 0.94; 95% CI, 0.79–1.11). In addition, subgroup analyses found that eczema significantly decreased the glioma risk in Europeans (OR, 0.73; 95% CI, 0.65–0.82) but not Australians (OR, 0.53; 95% CI, 0.14–2.02) or Americans (OR, 1.01; 95% CI, 0.69–1.46).

**Conclusion:**

Eczema may be considered as a potential protective factor of brain cancer in population aged over 16 years. However, this relationship requires verification using large-scale clinical data.

**Supplementary Information:**

The online version contains supplementary material available at 10.1186/s12885-022-10471-0.

## Introduction

Brain cancer is one of the most aggressive tumors owing to poor treatment effects [1, 2]. More than 90% of brain tumors occur in the brain parenchyma, with the remainder occurring in the meninges, spinal cord, and cranial nerves [3, 4]. Brain cancers are the primary cause of cancer-related deaths in children and adults worldwide. In 2016, 330,000 central nervous system cancer cases were reported globally, of which 227,000 patients died [5]. The reasons for high rates of brain tumor recurrence and mortality remain unclear. However, several have investigated the potential risk factors for brain tumors. For example, epidemiological studies have shown that air pollution, diet, allergies, medications, genetic predispositions, and some demographic characteristics (e.g., age, sex, and race) may increase the disease risk [6]. However, these factors require confirmation. Therefore, the need to identify new reliable risk factors for early brain tumor interventions is urgent.

Eczema is a common chronic cutaneous inflammatory disease affecting 10–30% of children and 2–10% of adults worldwide [7]. Some reports indicate that eczema affects the cancer risk, but the results are controversial [8, 9]. Therefore, this study performed a meta-analysis to assess the relationship between eczema and brain cancer to clarify the risk factors.

## Methods

### Search strategy

This meta-analysis was conducted following the Preferred Reporting Items for Systematic Reviews and Meta-Analysis (PRISMA) [10] and the Meta-analysis of Observational Studies in Epidemiology (MOOSE) [11] guidelines. We searched the PubMed and Embase databases for studies focusing on the relationship between eczema and brain cancers from inception until June 23, 2022. Supplementary Material 1 describes the specific search parameters. Two reviewers (LJ and ZY) independently performed a preliminary screening of relevant literature based on the titles and abstracts. Then, the full text of these papers was reviewed to identify those suitable for analysis. Differences in opinions were resolved through a discussion with the corresponding author. In addition, articles in the bibliographies of the papers mentioned above that met the screening criteria were also assessed for analysis potential.

### Selection criteria

The inclusion criteria were: a) human studies; b) observational studies including cohort and case-control study designs; c) published in English; and d) studies focusing on the relationship between eczema and the risk of brain tumors.

The exclusion criteria were: a) reviews, animal studies, abstracts, or conference proceedings; b) unpublished data or case reports; c) duplicate literature and duplicate data; and d) studies lacking specific data on exposure (eczema) and endpoints (brain tumors).

### Data extraction and quality assessment

The following relevant information was independently extracted by two reviewers (XSL and XYD) from the included studies: first author’s name, country of origin, publication year, study design, sample size, age, percentage of females, tumor type, number of patients, years of follow-up, and odds ratios (ORs) with 95% confidence intervals (CIs). For this meta-analysis, we defined eczema as the exposure factor and the occurrence of various brain tumors as the outcome. Data not directly obtainable from the text were extracted from the associated information (e.g., graphs). The corresponding authors were contacted to retrieve missing data.

Two authors (TYR and YWM) used the Newcastle-Ottawa Scale (NOS) [12] to evaluate the quality of the included studies based on three aspects: selection, comparability, and outcome assessments. The scores were summed, and studies with NOS scores of ≥5 were considered high quality [13].

### Statistical analyses

Stata software (version 12.0; Stata Corp. LLC, College Station, TX, USA) was used for the meta-analysis. The heterogeneity of the included papers was estimated using a homogeneity test (Q test) and the I^2^ value. A *P*-value of > 0.1 and I^2^ value of < 50% indicated acceptable heterogeneity, and then a fixed effects model was used to calculate pooled ORs and 95% CIs [14]; otherwise, the random effects model was used [15]. Subgroup analyses were performed to determine differences in the associations between eczema and brain tumors by age (≥16 years and <16 years) and study location (Australians, Americans, Europe and others). Sensitivity analyses were also performed by excluding one study at a time and then rerunning the analyses to calculate the effects for the remaining studies to determine if the pooled results were markedly affected by a single study. Finally, publication bias was evaluated using Begg’s funnel plot and Egger’s weighted regression test.

## Results

### Search results and study descriptions

**Fig. 1:**
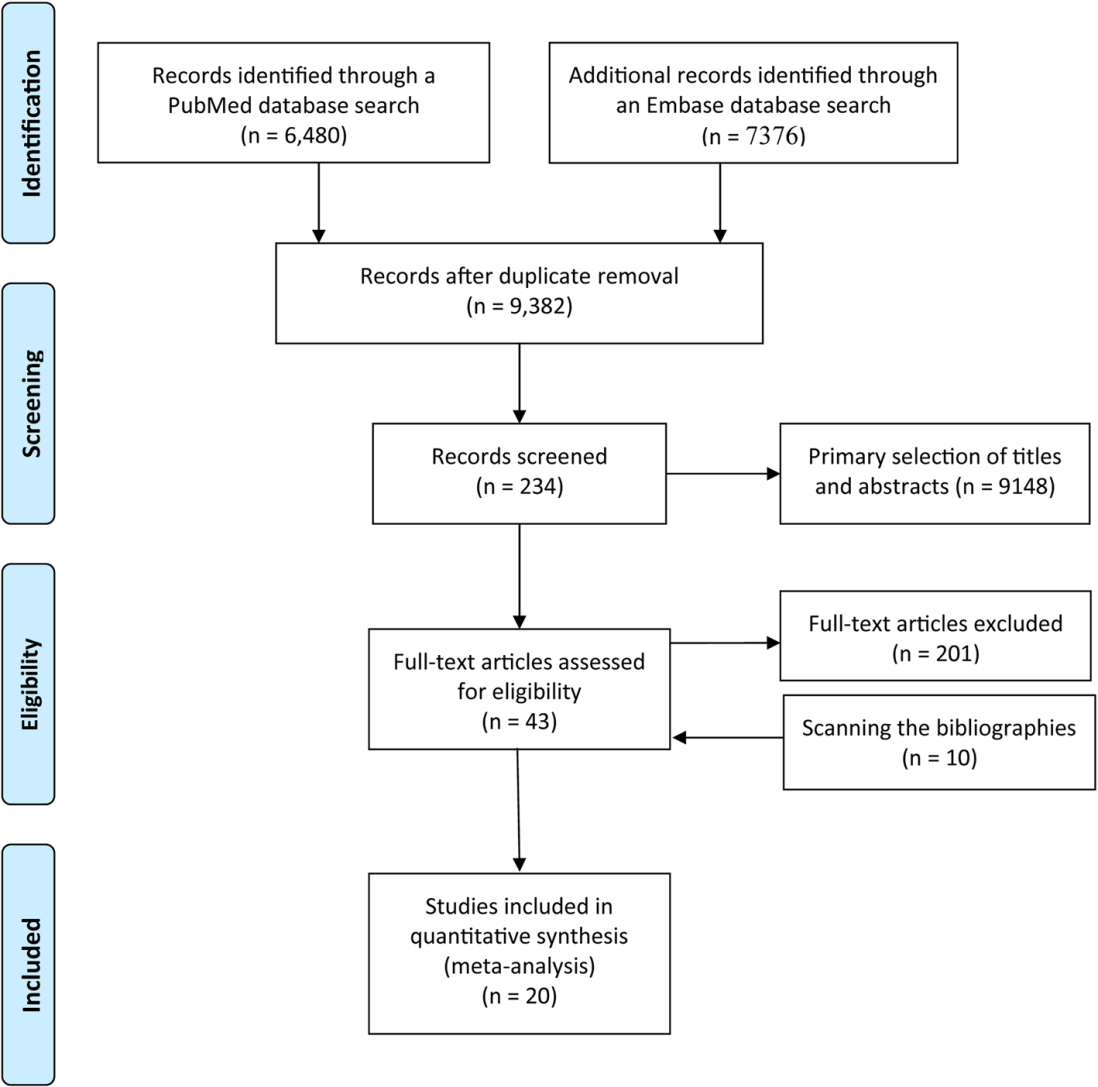
Flow diagram summarizing the pooled analysis phases

We retrieved 9382 records that included 5,117,222 patients. After screening the titles and abstracts, 9148 studies were removed. After assessing the full text of the remaining articles and relevant articles in their bibliographies, 20 studies [16–35] were included (Fig. 1). Of these, two studies [20, 35] were cohort studies, and 18 [16–19, 21–34] were case-control studies. The ages of the participants ranged from 1 to 99 years. All included studies had a NOS score greater than 5 and met the inclusion criteria; Table 1 presents these results.

**Table 1:** Basic characteristics of the included studies

Study, year	Study design	Region	Cancer	Sample size	Cases	Controls	Age range	Date range	NOS score
Ryan, 1992 [16]	Case-control	Australia	Glioma	527	110	417	25–74	1987.02–1990.03	7
Cicuttini, 1997 [17]	Case-control	Australia	Glioma	838	416	422	20–70	1987.7–1992.10	8
Schlehofer, 1999(M) [18]	Case-control	Other	Meningioma	1454	331	1123	20–80	1980–1991	6
Schlehofer, 1999(G) [18]	Case-control	Other	Glioma	3165	1178	1987	20–80	1980–1991	6
Brenner, 2002 [19]	Case-control	America	CNS tumors	1288	489	799	18–90	1994.6–1988.8	7
Schwartzbaum, 2003(G) [20]	Cohort	Europe	Glioma	14,535	NA	NA	42–81	NA	9
Schwartzbaum, 2003(M) [20]	Cohort	Europe	Meningioma	14,535	NA	NA	42–81	NA	9
Schwartzbaum, 2005 [21]	Case-control	Europe	Glioma	806	173	633	43–62	NA	8
Schoemaker, 2006 [22]	Case-control	Europe	Glioma	2681	965	1716	18–59	2000–2004	8
Schoemaker, 2007 [23]	Case-control	Europe	Meningioma	2191	475	1716	18–69	2001–2004	7
Wigertz, 2007 [24]	Case-control	Europe	CNS tumors	4836	1527	3309	≥18	2000.9–2004.2	8
Harding, 2008 [25]	Case-control	Europe	CNS tumors	6867	575	6292	2–15	1991–1996	8
Berg-Beckhoff, 2009(G) [26]	Case-control	Europe	Glioma	1098	366	732	30–69	2000.11–2003.11	8
Berg-Beckhoff, 2009(M) [26]	Case-control	Europe	Meningioma	1143	381	762	30–69	2000.11–2003.11	8
Il’yasova, 2009 [27]	Case-control	America	Glioma	565	388	177	≥18	2003.8–2008.4	7
Schoemaker, 2009 [28]	Case-control	Europe	Pituitary tumor	929	299	630	18–59	2000.12–2005.2	8
Roncarolo, 2012 [29]	Case-control	America	CNS tumors	16	6	10	0–14	1980–1999	9
Turner, 2013 [30]	Case-control	Other	Acoustic neuroma	199	34	165	30–59	2000-–004	8
Amirian, 2016 [31]	Case-control	Other	Glioma	8019	4270	3749	18–80	NA	7
Lupatsch, 2018 [32]	Case-control	Europe	CNS tumors	3188	469	2719	1–14	NA	8
Pouchieu, 2018(G) [33]	Case-control	Europe	Glioma	777	253	524	≥16	2004–2010	8
Pouchieu, 2018(M) [33]	Case-control	Europe	Meningioma	627	206	421	≥16	2004–2010	8
D’Arcy, 2019 [34]	Case-control	America	CNS tumors	1,844,575	15,205	100,000	66–99	1992–2013	7
Mansfield, 2020(U) [35]	Cohort	Europe	CNS tumors	2,711,745	NA	NA	24.9–60.7	1998.2–2016.3	8
Mansfield, 2020(D) [35]	Cohort	Europe	CNS tumors	490,618	NA	NA	1.7–21.1	1982.2–2016.6	8

*CNS* Central nervous system, *NA* not mentioned, *NOS* Newcastle-Ottawa Scale

### Eczema and brain cancer risk

**Fig. 2:**
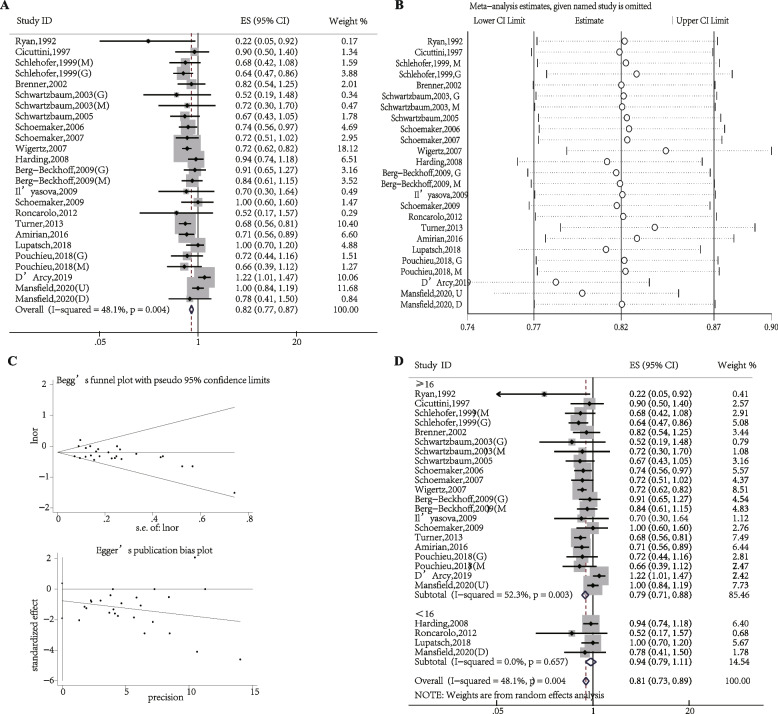
The association between eczema and brain cancer risk. **A** Forest plot of the estimated effects of eczema on brain cancer risk. **B** Sensitivity analysis conducted by recalculating the pooled results of the primary analysis following the exclusion of one study per iteration. **C** Begg’s and Egger’s tests indicate the lack of publication bias among such studies. **D** Subgroup analysis of the estimated effects of eczema on brain cancer risk. CI, Confidence interval; ES, Effect size

Eczema was significantly associated with a decreased brain cancer risk in the analysis with all 20 studies [16–35] (OR, 0.82; 95% CI, 0.77–0.87; Fig. 2A). Insignificant heterogeneity greatly enhanced the reliability of the results (*P* = 0.004, I^2^ = 48.1%; Fig. 2A). The sensitivity analysis indicated that excluding each study in turn all studies were distributed between the upper and lower 95% CIs with the estimation line, and excluding any one study did not alter the overall combined results (Fig. 2B). Publication bias, assessed by Begg’s rank correlation and Egger’s linear regression, did not identify publication bias among the studies (Begg’s: *P* > |z| = 0.362; Egger’s: *P* = 0.183, 95% CI: − 1.900–0.384; Fig. 2C).

Next, to test this hypothesis that the brain cancer risk might be influenced in different aged eczema patients, subgroup analyses based on age were performed; we identified a strong association between eczema and brain cancer risk in those aged over 16 years (OR, 0.79; 95% CI, 0.71–0.88) but no association for those under 16 years old (OR, 0.94; 95% CI, 0.79–1.11; Fig. 2D).

### Eczema and glioma risk

**Fig. 3:**
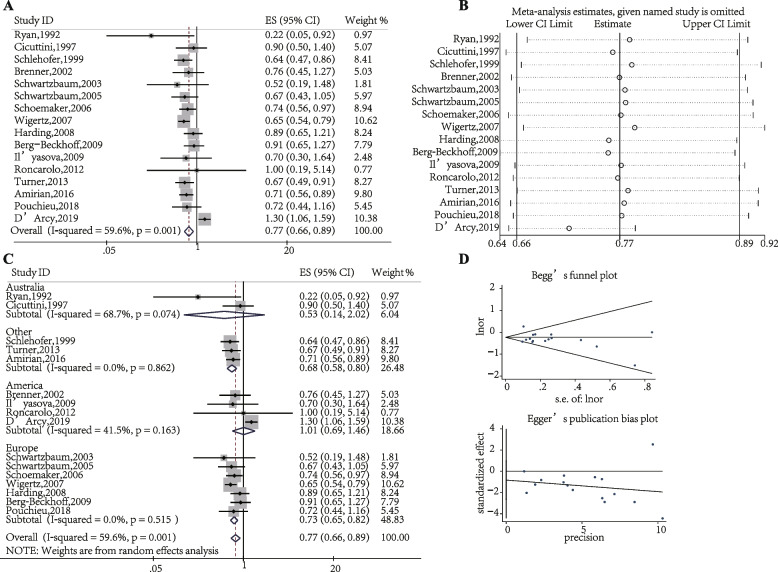
The association between eczema and glioma risk. **A** Forest plot of the estimated effects of eczema on glioma risk. **B** Sensitivity analysis. **C** Subgroup analysis of the estimated effects of eczema on glioma risk. **D** Begg’s and Egger’s tests indicate the lack of publication bias among such studies. CI, Confidence interval; ES, Effect size

Eczema significantly decreased the glioma risk (OR, 0.77; 95% CI, 0.66–0.89; Fig. 3A); the analysis included 16 studies [16–22, 24–27, 29–31, 33, 34]. The sensitivity analysis confirmed that the meta-analysis was robust (Fig. 3B). However, significant heterogeneity was observed (*P* = 0.001, I^2^ = 59.6%; Fig. 3A). Subgroup analyses were performed based on the study region, indicating that eczema significantly decreased the glioma risk for Europeans (OR, 0.73; 95% CI, 0.65–0.82), and the heterogeneity disappeared (*P* = 0.515, I^2^ = 0.0%). However, the relationship between eczema and glioma risk was statistically insignificant for Australians (OR, 0.53; 95% CI, 0.14–2.02; I^2^ = 68.7%) and Americans (OR, 1.01; 95% CI, 0.69–1.46; I^2^ = 41.5%) (Fig. 3C). Therefore, the study region may be a source of heterogeneity. Publication bias was not observed among the studies (Begg’s: *P* > |z| = 0.685; Egger’s: *P* = 0.344, 95% CI: − 2.635–0.983; Fig. 3D).

### Eczema and meningioma risk

**Fig. 4:**
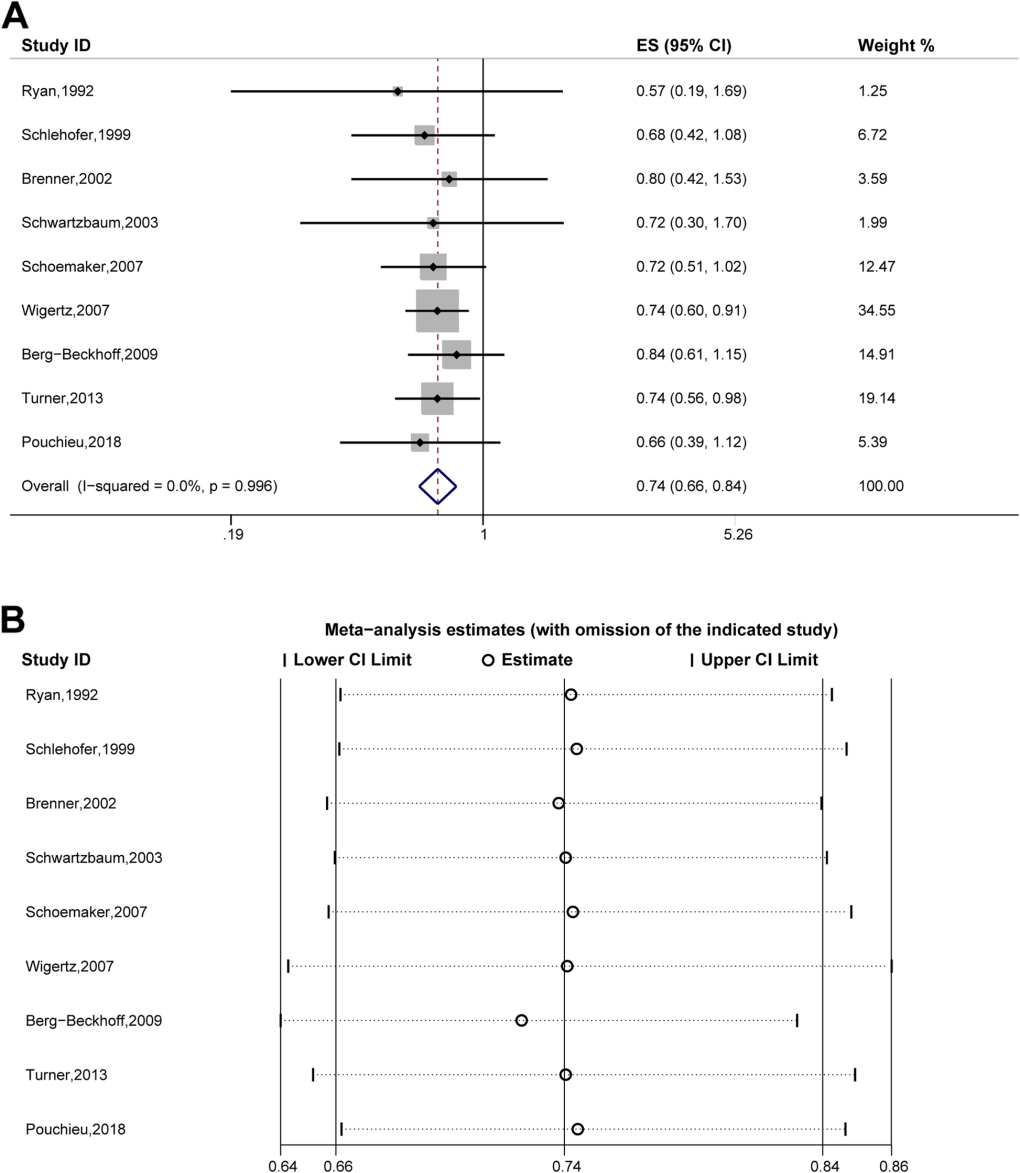
The association between eczema and meningioma risk. **A** Forest plot of the estimated effects of eczema on meningioma risk. **B** Sensitivity analysis. CI, Confidence interval

Eczema was significantly associated with a decreased risk of meningioma (OR, 0.74; 95% CI, 0.66–0.84); the analysis included nine studies [16, 18–20, 23, 24, 26, 30, 33]. Heterogeneity was not observed (*P* = 0.996, I^2^ = 0.0%; Fig. 4A), and excluding individual studies did not alter the overall results (Fig. 4B), indicating that these results were reliable.

### Eczema and acoustic neuroma risk

**Fig. 5:**
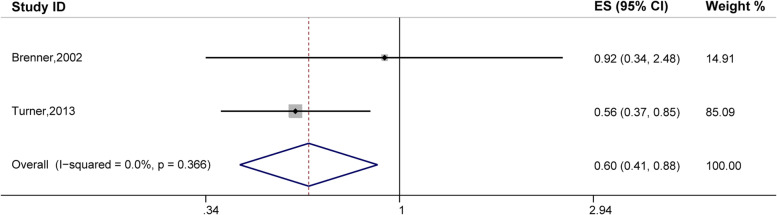
Forest plot of the estimated effects of eczema on acoustic neuroma risk. Confidence interval

Eczema was significantly associated with a decreased risk of acoustic neuroma (OR, 0.60; 95% CI, 0.41–0.88); the analysis included two studies [19, 30]. There was no heterogeneity (*P* = 0.366, I^2^ = 0.0%; Fig. 5).

## Discussion

This meta-analysis aimed to advance our understanding of the association between eczema and brain cancer by analyzing 20 related papers. The strong correlation between eczema and the reduced risk of brain cancer was observed in people over the age of 16, but not in the age of 16, which indicated that eczema may be considered as a potential protective factor when assessing the risk of brain cancer in population aged over 16 years. At the same time, eczema was significantly associated with a decreased risk of glioma, meningioma, and acoustic neuroma. However, the relationship between eczema and the risk of glioma differed regionally. Specifically, eczema significantly decreased the glioma risk in the European subgroup without heterogeneity but not in the Australian and American subgroups.

Many previous studies have investigated associations between allergic diseases and the risk of various cancers [29, 36, 37] but with inconsistent results. For instance, Hwang et al. reported complex and site-specific relationships between allergic diseases (e.g., eczema, allergic rhinitis, and asthma) and cancer risk [8]. Unexpectedly, the incidence of brain cancer was higher in patients with eczema [36]; however, the sample size was too small to be reliable. Conversely, another study reported that children with eczema did not have a lower risk of brain tumors (OR, 0.52; 95% CI, 0.17–1.57) [29], which supported our subgroup analysis results. Additionally, a recent study demonstrated that eczema, an allergic disease that increases immune surveillance, may be related to the low risk of many cancers, supporting our results [37]. Unlike our meta-analysis, these reviews preferentially pooled crude estimates and included broader criteria, focusing on the effects of all allergic diseases on the incidence of several cancers; nonetheless, their results were consistent with ours.

Numerous studies have reported inverse associations between eczema and several cancer sites [31, 38, 39]. However, how eczema affects the incidence of tumors remains debatable. Many researchers agree that the severity of eczema varies widely, and only the most severe cases are identifiable [34]. However, in our study, eczema was associated with a reduced risk of brain cancer. Some theories have been proposed to support this conclusion. Specifically, the effects of allergic diseases on the risk of glioma are close to consensus. One hypothesis is that since eczema is an allergic disease, it may indicate a high state of immune surveillance [40–42]. Consequently, the overactive immune system suppresses abnormal cell growth or proliferation. However, the mechanisms by which the enhanced immune surveillance hypothesis helps suppress tumor growth remain unclear. An additional explanation for this protective effect is that allergies are a defense mechanism against infestation by large parasites and environmental toxins [43]. As a result, individuals with stronger allergic reactions excrete and eliminate environmental toxins or carcinogens more efficiently throughout their lives than those without strong reactions. Interestingly, the association between eczema and glioma risk varied in the European, Australian, and American regions. However, the mechanisms underlying these differences have not been elucidated. Studies have shown that the glioma incidence rate varies globally, with the highest rates observed in America, Australia, and Northern Europe [44]. Therefore, the risk of glioma is closely related to the region, descent, and race or ethnicity [45].

This study had some limitations. First, some of our results had heterogeneity, which might affect their reliability. However, heterogeneity did not exist between eczema and meningiomas and acoustic neuromas; the relationship between eczema and glioma was an exception. Therefore, heterogeneity minimally affected our conclusions. Second, our study population spanned a wide age range, but data limitations prevented us from investigating the effects of age on the results; we could only evaluate associations. Finally, we did not find publication bias in our analyses because researchers only reported positive results. Therefore, we inevitably could not assess the effects of unpublished studies with negative results. Nonetheless, our study included many patients and individually eliminated studies to evaluate sensitivity, making our results more comprehensive and reliable.

## Conclusion

Eczema was significantly inversely associated with the risk of brain cancer, including glioma, meningioma, and acoustic neuroma. Therefore, eczema is a promising predictor of brain tumor risk. However, the study location affects this relationship. Therefore, these results require validation in large randomized controlled trials.

## Supplementary Information

**Additional file 1. **Supplementary Material.

## Data Availability

The original contributions presented in this study are included in the article and supplementary material. Further inquiries can be directed to the corresponding author.
